# Biological Mechanisms of Atropine Control of Myopia

**DOI:** 10.1097/ICL.0000000000000677

**Published:** 2020-01-02

**Authors:** Aradhana Upadhyay, Roger W. Beuerman

**Affiliations:** Singapore Eye Research Institute (A.U., R.W.B.), Singapore; Ophthalmology and Visual Sciences Academic Clinical Program (A.U., R.W.B.), Duke-NUS Medical School, Singapore; and Duke-NUS SRP Neuroscience and Emerging Infectious Diseases (R.W.B.), Duke-NUS Medical School, Singapore.

**Keywords:** Myopia, Myopia control, Biological mechanisms in myopia, Atropine, Atropine action in myopia

## Abstract

Myopia is a global problem that is increasing at an epidemic rate in the world. Although the refractive error can be corrected easily, myopes, particularly those with high myopia, are susceptible to potentially blinding eye diseases later in life. Despite a plethora of myopia research, the molecular/cellular mechanisms underlying the development of myopia are not well understood, preventing the search for the most effective pharmacological control. Consequently, several approaches to slowing down myopia progression in the actively growing eyes of children have been underway. So far, atropine, an anticholinergic blocking agent, has been most effective and is used by clinicians in off-label ways for myopia control. Although the exact mechanisms of its action remain elusive and debatable, atropine encompasses a complex interplay with receptors on different ocular tissues at multiple levels and, hence, can be categorized as a shotgun approach to myopia treatment. This review will provide a brief overview of the biological mechanisms implicated in mediating the effects of atropine in myopia control.

Myopia is the most common refractive disorder worldwide and has become a leading cause of blindness.^1^ A systemic review and meta-analysis of 145 different studies from around the world involving 2.1 million participants suggests that approximately 23% and 2.7% of the world population had myopia and high myopia in 2000, respectively.^2^ It has been predicted that myopia prevalence will increase to nearly 50% by 2050 affecting 4.8 billion people in the world, which means that one in every two children will become myopic in approximately 30 years from now. The incidence of myopia varies according to ethnicity and geographical location, affecting approximately 85% of urban population in East Asia, especially in Taiwan^3,4^ and Singapore,^5^ and 44% in the United States.^6^ Interestingly, the prevalence is much lower in underdeveloped areas in the world such as rural Mongolia (5.8%)^7^ and Pokhara, Nepal (4.05%).^8^ Myopia has been associated with increased risk of comorbidities such as retinal detachment, macular degeneration, foveoschisis, early-onset glaucoma, cataract,^9^ and even vision loss,^10^ such that the risk is greater at higher degrees of myopia. The yearly incidence of retinal detachments increases with the degree of myopia (0.015% in >4.74 D myopia, 0.07% in >5.00 D myopia, and 3.2% in >6.00 D myopia).^11^ Even the risk of developing macular choroidal neovascularization increases with the level of myopia, with the likelihood being as high as 2-fold between 1.00 to 2.00 D myopia, 4-fold between 3.00 to 4.00 D myopia, and 9-fold for 5.00 to 6.00 D myopia.^12^ The economic burden of myopic refractive error was estimated to be US$202 billion per annum, with at least $3.8 billion spent as direct costs on myopia correction annually.^13,14^ These factors pose key challenges in managing myopia, prevention of which has become a major public health issue.^3^

Myopia is characterized by excessive axial elongation because of an increase in the vitreous chamber depth of the eyes, which causes light from distant objects to focus in front of the retina, leading to formation of blurred images. In other words, there is a mismatch between the focal length and the axial length (AL) of the eye in myopia, with the latter being too long for the refractive power of the lens and cornea. Myopia is a complex multifactorial disorder regulated by interactions between environmental and genetic risk factors^15^ and occurs as a result of failure to achieve or maintain the normal process of emmetropization, which is essentially endogenous to the eye.^16^ Myopia generally develops during childhood, during the school years, and its progression gradually stabilizes after adolescence for most individuals. Several epidemiological and animal studies conducted over the past 4 decades have investigated the possible causes underlying the development of myopia. A complexity of conditions including environmental factors such as time spent outdoors,^17^ near work,^5^ prolonged intense education,^18^ and urbanization^19^ play an important role in the development of myopia in school-going children. Nevertheless, despite a plethora of myopia research, the molecular/cellular mechanisms underlying the development of myopia are not well understood, preventing the search for the most effective pharmacological control. Consequently, there is no established way to prevent the onset of myopia, totally stop the progression, or reverse the progression. Most existing strategies to control the epidemic of myopia, thus, aim for effectively managing the disorder—by delaying its onset or slowing down myopia progression in the actively growing eyes of children through increased time spent outdoors^20^ and decreased duration of near work,^21^ by pharmacological interventions^22^ and other optical strategies (such as bifocal and multifocal lenses, progressive addition lenses, soft bifocal contact lenses, and orthokeratology),^23,24^ and refractive correction clinically by prescription lenses, contact lenses, or surgery.^25^

## DECREASING PROGRESSION OF MYOPIA

Myopia control using pharmaceutical interventions has been reported to be most effective in comparison with other strategies.^26,27^ Anticholinergics are a class of drugs which block the action of acetylcholine at the muscarinic receptors (MRs) on structures with parasympathetic innervation and smooth muscles. Acetylcholine plays an important role in the developing retina^28^ and regulates the growth of the eye.^29^ Several drugs from this class have displayed variable efficacy to slow myopia progression in the Cochrane database systemic review of 2011.^30^ However, only atropine sulfate^29,31–33^ (a nonselective, broad muscarinic acetylcholine receptor antagonist) and pirenzepine^34,35^ (an antagonist with selectivity for M1R subtype) have shown clinical effectiveness in rigorous trials. Daily topical application of atropine at 1% or lower concentrations reduced myopia progression in children in a dose-dependent manner in several studies, with significant reductions in change in cycloplegic refraction (spherical equivalent [SE]) and AL elongation by as much as 80% and 95%, respectively, at the dose of 1%.^29^ Likewise, pirenzepine at 2% also demonstrated an efficacy of 50% in slowing myopia progression with the requirement of dosing twice a day instead of once as in the case of atropine.^35^ Nonetheless, further testing of pirenzepine in clinical trials has been suspended perhaps because of regulatory and economic constraints.^36^ Thus, atropine is the currently available anticholinergic drug for off-label use in myopia treatment with demonstrated consistent efficacy.^26,30^ Myopia progression, as determined by change in the cycloplegic refraction, was reduced by 75%, 70%, and 60% at 0.5%, 0.1%, and 0.01% atropine, respectively, in comparison with the historic placebo group after two years of treatment in phase 1 of the ATOM2 study. Axial length elongation, on the other hand, was decreased by 29% and 26% at 0.5% and 0.1% atropine, respectively, with no observed effect of 0.01% atropine on curtailing AL elongation.^37^ The more recent placebo-controlled low-concentration atropine for myopia progression (LAPMP) study showed that although myopia progression was minimized by 67%, 43%, and 27%, the AL elongation was reduced by 51%, 29%, and 12% in the 0.05%, 0.025%, and 0.01% atropine groups, respectively, after 1 year of treatment.^33^ Although direct comparisons cannot be made between these studies given the differences in their strategies and trial duration, it must be noted that in both the studies, only the mean change in SE was significantly different between 0.01% atropine-treated and the placebo groups, whereas AL elongation was nonsignificant. Also, AL elongation in the 0.01% atropine group at 2 years was significantly greater in comparison with other groups treated with higher atropine concentrations (0.1% and 0.5%).^37^ However, the intergroup differences were small and clinically insignificant. This might create some uncertainty over the effectiveness of 0.01% atropine for myopia control, given the importance of axial elongation in myopia progression, but we must be careful about jumping to conclusions and also consider the long-term effects of atropine after stopping the treatment.^38^ Unfortunately, atropine usage is associated with several ocular side-effects such as mydriasis (pupil dilation), photophobia, glare, local allergic response, loss of accommodation, and near vision (cycloplegia), which wear out eventually with the cessation of atropine treatment. These side-effects are more common at higher concentrations and seem to be dose-dependent.^31^ In fact, the incidence of photophobia was 100% in children receiving 1% atropine, leading to a high dropout rate of 16% to 58% from a study.^39^ On the contrary, the percentage of participants experiencing photophobia was reduced to 22% and 7% at lower atropine concentrations of 0.5% and 0.25%, respectively, and none at 0.1% atropine.^40^ Likewise, only 7% of participants on 0.01% atropine experienced photophobia and requested photochromatic (tinted) glasses in the ATOM2 study.^31^ Besides the temporary side-effects associated with atropine treatment, there is a more pronounced rebound phenomenon, in which myopia returns at a faster rate in the treated, myopic eyes than in the untreated eyes on cessation of treatment. Even the observed rebound effect was greater at higher concentrations of atropine, with 68% (on 0.5% atropine), 59% (on 0.1% atropine), and only 24% (on 0.01% atropine) of participants experiencing greater than 0.50 D increase in myopia after stopping the treatment (washout phase).^31^ In reality, during the washout phase, the change in mean SE and AL elongation was the least (*P*<0.001) in the 0.01% atropine group,^38^ whereas myopia progression continued at a steady pace in groups previously receiving 0.1% and 0.5% atropine, slowing only when 0.01% atropine was restarted in phase 3. This suggests that atropine, particularly at higher concentrations, could induce complex, long-lasting biochemical changes in the mechanisms regulating eye growth. Gradual tapering of atropine over time could possibly reduce the rebound effect, but this has not been studied in detail. Taken together, over the period of 5 years, the 0.01% atropine group showed the lowest overall myopia progression with least change in SE and AL elongation values and minimal visual side-effects among all the treatment groups. Long-term atropine usage could be associated with increased intraocular pressure and possibly glaucoma. Investigating this, clinical studies have reported no impact of atropine treatment on ocular hypertension in children,^41,42^ and that the risk of atropine-induced glaucoma is as low as 0.005%.^43^ Thus, so far, 0.01% atropine has shown the best therapeutic index (appropriate risk:benefit ratio) with overall better effectiveness and more modulated and sustained effect than higher doses.^31,38^ Although 0.05% atropine has been reported to show improved myopia-suppressive effects than 0.01%, with no adverse effect on vision-related quality of life over the period of 1 year,^33^ more information is required on the efficacy of 0.05% atropine in the longer term, particularly its effect on myopia rebound, before it can be validated and imbibed in clinical practice regularly. The efficacy of 0.01% atropine in myopia control has also been replicated outside Asia in an ethnically diverse group of children in the United States; however, this was determined from noncycloplegic refraction data.^32^ In fact, 0.01% atropine formulation has even been commercialized as Myopine. This product is now available in Singapore and Malaysia on an approved, named-patient basis and has been licensed in 15 countries across Europe and Asia to date.^44^ Yet, the exact mechanisms mediating atropine action in slowing myopia progression are unclear and remain a matter of speculation.

## CELLULAR RECEPTORS ARE INVOLVED

Pharmacologically, atropine acts as a reversible competitive antagonist with an affinity for all the five subtypes of acetylcholine MRs (MR1–MR5) and thus has been presumed to exert its myopia-protective effect mainly through the MRs. The MRs belong to the superfamily of G protein–coupled receptors (GPCRs) and have both a neuronal and non-neuronal presence in the eye. Muscarinic receptors are widely distributed in different ocular tissues in mammals. They are found in cornea, iris, ciliary body, and ciliary muscles,^45^ epithelium of crystalline lens,^46^ retina (in amacrine cells), retinal pigment epithelium (RPE),^47^ choroid, and sclera (in scleral fibroblasts [SF]).^48,49^ However, several studies have reported potential off-target direct and indirect interactions of atropine at other non-MRs such as α_2A_-adrenergic receptors (αAR),^50^ ɣ-aminobutyric acid receptors (GABA-R),^51^ and receptor tyrosine kinases (RTKs)^48^ in different ocular tissues. Both αAR and GABA-R are well-known members of the GPCR family. Epidermal growth factor receptor (EGFR) is a member of the ErbB family of RTKs and regulates cellular proliferation of primary SF through intracellular signaling pathways involving the classical mitogen-activated protein kinase pathway.^48^ Atropine has been shown to reduce EGFR activity in mouse primary SF in a dose-dependent manner.^48^ Thus, atropine is a shotgun approach to myopia treatment. Corroborating this conjecture, a recent study analyzing the ocular pharmacokinetics of 1% topical atropine in rabbits showed that atropine has good bioavailability in most of the ocular tissues with detectable concentrations of twofold greater than its binding affinity (0.4–0.7 nM) at 3-day postdrug instillation.^52^ Although the penetration of atropine was greater in the anterior tissues (concentration gradient: highest in the conjunctiva, lowest in the lens) at 5 hr after instillation, an increased binding to posterior tissues was observed at 24 hr, with the reversal of the initial concentration gradient (highest in the posterior sclera, followed by the retina). In addition to its antagonistic properties at different biological receptors, atropine can also act as an inverse agonist at MR3, and probably at all the other MR subtypes, since it was shown to reduce the basal receptor activity in vitro in a concentration-dependent manner.^53^

**FIG. 1.**
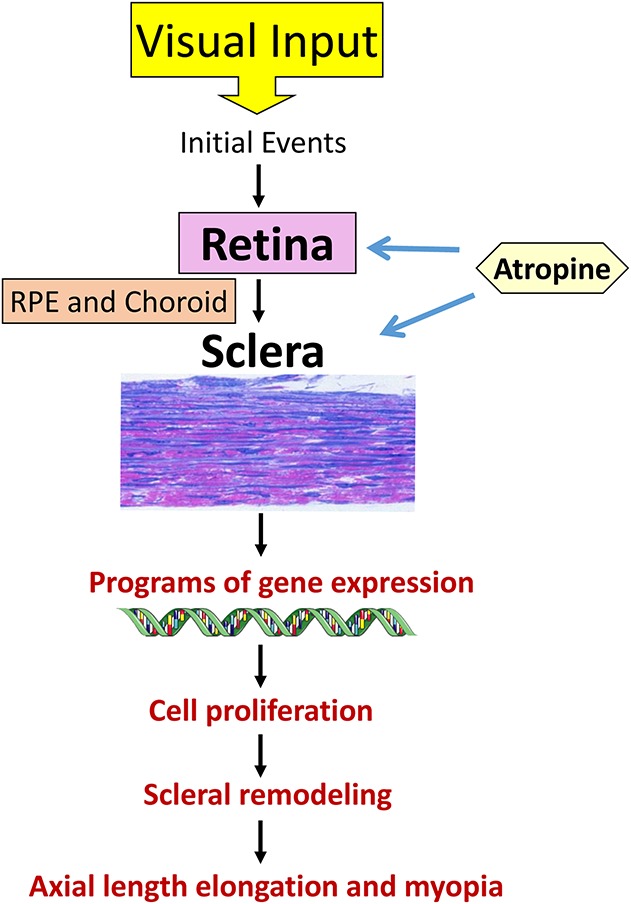
Schematic representation of signal cascade mediating myopia development in animal models. The available evidence points to an interaction between the retina and sclera in the development of myopia. However, this is largely from animal model studies where visual cues such as optical defocus imposed by lens wear predominantly drive axial elongation. These visual inputs initiate certain molecular events in retina. The growth-regulatory signals are then relayed to the sclera through RPE and choroid to facilitate programs of gene expression, cell proliferation, and scleral remodeling, which subsequently lead to axial length elongation causing myopia. We have little idea as to the situation in humans, where genetic and environmental factors may also play a role in myopia induction and development. Moreover, we do not know the nature of intertissue communication that may be quite complex in humans and even change in different stages of myopia development. Atropine targets biological receptors in both the retina and sclera, to curtail myopia progression. RPE, retinal pigment epithelium.

Atropine was initially used for myopia control because of historical reasons since excessive accommodation by the eye was hypothesized to be responsible for myopia and atropine causes cycloplegia (temporary loss of accommodation/ability to focus on near and distant objects) by paralyzing the smooth ciliary muscle temporarily. However, later animal studies implicated nonaccommodative mechanisms in the cause of myopia and showed that eyes unable to accommodate, either due to lesioning of the Edinger–Westphal nucleus^54^ (the parasympathetic innervation of the iris sphincter muscle and the ciliary muscle) or sectioning of the optic nerve,^55^ also compensated to the imposed hyperopic defocus and developed myopia. In addition, atropine was found to reduce experimental form-deprived myopia (FDM) in chick eyes^56^ that possess acetylcholine nicotinic receptors on their straited intraocular muscles instead of MRs.^57^ Thus, there has been a paradigm shift of focus on investigating nonaccommodative mechanisms in myopia development. Concordantly, several biological mechanisms such as dysfunction of retinal signaling pathways in response to environmental cues,^58–60^ role of RPE in relaying ocular growth regulatory signals from the retina to sclera through choroid,^61–63^ reduced choroidal thickness,^64,65^ and increased scleral thinning due to remodeling of the extracellular matrix of the sclera^66,67^ have been thoroughly investigated to comprehend the causes of myopia (Fig. 1). Atropine has been observed to modulate these biological mechanisms and target mainly the retina and sclera, although effects of atropine on the choroid^68,69^ and RPE^70^ have also been reported.

### Role of the Retina

Retina is the light-sensitive, sensory layer lining the inner surface of the back of the eye. Intravitreal injection of atropine increased dopamine release and the concentration of its metabolite DOPAC in the chick retina.^71^ Dopamine is a neurotransmitter that facilitates interneuron signaling and plays a crucial role in ocular growth and myopia development.^72,73^ Dopamine agonists such as apomorphine inhibit myopia progression in both the myopiagenic animal models of form-deprived^74^ and lens-induced myopia (LIM).^75^ Atropine also reduced protein levels of GABA transporter-1 (GAT-1) and to a lesser extent GAT-3 that were elevated in the mouse retina with LIM, thereby suggesting the involvement of GABAergic signaling in the antimyopic effects of atropine in mouse eyes.^51^ The GABAergic transmission in the neural retina plays a pivotal role in the modulation of eye growth and refractive development in animals.^76^ GABA transporters, on the other hand, terminate the GABAergic transmission by facilitating its reuptake into presynaptic neurons and surrounding glial cells and removing GABA from the synaptic cleft.^77^ Interestingly, atropine was shown to restrain FDM in chicks with destroyed cholinergic amacrine cells, MRs, and choline acetyltransferase (ChAT, an enzyme that synthesizes acetylcholine) activity in the retina in a dose-dependent manner.^78^ This suggests that retinal MRs do not mediate the growth-inhibiting effects of atropine at least in FDM and indicated the involvement of MRs in other ocular tissues.

### Role of the Sclera

Sclera is a specialized fibrous connective tissue that forms the protective outer layer of the eye. Excessive axial elongation of the eye causes refractive error and myopia development and can be attributed to scleral thinning due to increased scleral tissue remodeling. Certain molecular and biochemical events take place in the sclera during this remodeling process, which eventually cause structural and biomechanical changes in the tissue, resulting in weakened extracellular matrix and significant scleral tissue thinning (reviewed elsewhere in detail^79^). Inhibition of myopia progression by atropine can also occur at the scleral site. This conjecture is supported by evidences showing that atropine decreased proliferation of mouse SF in vitro,^48^ increased the thickness of scleral fibrous layer in the myopic eyes from both chick^80^ and mouse^67^ models, reduced extracellular matrix production by decreasing glycosaminoglycan (GAG) synthesis both in the whole sclera and in isolated scleral chondrocytes from form-deprived eyes in chicks,^81^ and upregulated the mRNA levels of MR1, MR3, and MR4 while downregulating MR2 and MR5 mRNA levels in the sclera from mouse eyes with LIM.^67^

### Role of Retinal Pigment Epithelium and Choroid

Retinal pigment epithelium is a monolayer of highly specialized pigmented cells, which forms a barrier between the neural retina and vascular choroid, and plays a very important role in maintaining retinal homeostasis. Retinal pigment epithelium has been shown to relay the growth regulatory signal from the retina to choroid and sclera and modulate scleral growth and alter scleral GAG content in vitro.^61,62^ Coculturing of ex vivo RPE (from LIM chick eyes) with primary SF (from nonmyopic eyes) increased the cell proliferation of SF and decreased GAG content in vitro.^61^ By contrast, there was no effect of ex vivo retina and primary SF coculture on the latter's DNA or GAG content, while choroid and SF coculture increased the GAG content only. This suggests that the growth regulatory defocus signals, although originating in retina, are most likely contained in the RPE and possibly in the choroid to some extent. Choroid is the vascular layer of the eye and supplies oxygen and nourishment to the outer retina. Choroid modulates its thickness mechanistically to adjust the retina to the focal plane of the eye in response to the imposed optic defocus (choroidal accommodation) and hence plays an active role in emmetropization.^63^ Atropine inhibited the development of myopia and rapidly induced transient choroidal thickening in LIM model of chicks.^68^ Likewise, atropine eliminated choroidal thinning induced by hyperopic defocus signals in myopic human eyes without changing baseline choroidal thickness.^69^ Both the RPE (basal surface) and choroid secrete a variety of growth factors including transforming growth factor (TGF-β) and basal fibroblast growth factor (bFGF). Atropine has also been shown to modulate the expression and activity levels of these growth factors (different isoforms) in vitro. Atropine inhibited the expression and secretion of TGF-β2 by blocking MRs in RPE cells.^82^ Atropine also decreased TGF-β1 activity levels and increased bFGF2 activity levels in primary mouse SF in a dose-dependent manner.^48^ Transforming growth factor-β1 stimulates collagen synthesis by primary SF in vitro in a dose-dependent manner and thus has been implicated in the regulation of scleral remodeling during myopia development.^83^ bFGF2, on the other hand, activates Ras/mitogen activated protein kinase-mediated signaling in mouse SF^48^ and stimulates SF proliferation.^84^

## FUTURE CONSIDERATIONS

Atropine at 0.01% seems to be an effective, low-cost medication to slow myopia progression in the growing eye. Nonetheless, this outcome is mainly based on its sustained impact on restraining changes in refraction, with lesser effect observed on inhibiting AL elongation. The detailed mechanisms and the exact site of action of atropine also remain elusive. Further to this, muscarinic mechanisms have not been per se studied in the development of myopia. Although the side-effects observed at 0.01% atropine are minimal and apparent for short term, in the 0.01% group of ATOM2 study, 24% of participants were subjected to myopia rebound, 7% experienced mild side-effects that were not severe enough to prompt a discontinuation, and 9.3% responded poorly to the treatment since they had greater than 1.5 D myopia progression over 2 years of initial treatment. The percentages of “poor responders” were 6.4% and 4.3% in 0.1% and 0.5% atropine groups, respectively. Similarly, in other studies, 11% of children in the 0.5% atropine group had greater than 0.75 D myopia increase per year,^85^ whereas 33%, 17%, and 4% of children belonging to 0.1%, 0.25%, and 0.5% atropine groups, respectively, showed greater than 1 D increase of myopia per year in comparison with 44% in the control group.^40^ Another study found 45% of participants to be “poor responders” to 0.05% atropine with myopia progression of greater than 0.5 D over 6 months.^86^ However, when switched to 0.1% atropine, only 20% progressed further by greater than 0.5 D per year in comparison with 100% in the control group over 4.5 years of follow-up. Even when treated at 1% atropine, 12% of children at 1 year continued to progress by greater than 0.5 D myopia per year and were likely to be younger, more myopic, and have two myopic parents.^87^ The treatment strategy for such poorly responding patients remains obscure.

Several studies, including those on the actively growing eyes of children belonging to the similar age group as in the atropine trials (6–12 years), have shown that the distribution of refraction changes with age^88,89^ and indicated the presence of two different Gaussian subpopulations, with distinct patterns of refraction and AL distribution possibly due to differences in their etiologies, in adult populations.^90^ Although most population possesses emmetropic eyes during early childhood (6 years) with leptokurtotic distribution of refraction represented by narrow peak, there is a very small subpopulation with myopic refraction that fail to undergo the initial phase of emmetropization (indicating primary homeostatic failure) or show a delay in achieving early emmetropization (poor emmetropizers).^88^ As the eyes grow over time, by 11 to 12 years of age, a significant proportion of the population remain close to emmetropia (indicating regulated growth), but the spread of refraction data increases, and there is a shift toward negatively skewed distribution, suggesting the presence of another subset that begins to drift in the direction of myopia owing to dysregulated eye growth from the inability to maintain the emmetropic state (secondary homeostatic failure). Interestingly, the distribution of ocular biometry (AL and refraction) becomes bigaussian, with essentially two subpopulations existing in the adult population (20–70 years): an emmetropized subset with a sharp peak at emmetropia and less variability in the data and a dysregulated subgroup with a broader peak/mode, increased data spread/variability, and a myopic mean.^90^ Thus, myopes begin to become myopic either due to primary (a very small percentage though) or secondary homeostatic failures and therefore warrant a need for intervention to regulate their eye growth. Further to this, not all the eyes will emmetropize to the same extent, and some with low myopic refraction will fall within the tail of emmetropic refraction distribution (poor emmetropizers). It would, then, be expected that interventions such as atropine would have different effects on myopes belonging to these two groups: poor emmetropizers and those with failed homeostatic mechanisms, which can be determined from their individual myopia progression rates. In particular, atropine might have little effect on the poor emmetropizers, that perhaps overlap with the “poor responders” category to a certain extent. Furthermore, the possibility of any intervention exacerbating the extent of myopia in this group also cannot be negated.

There are concerns as well regarding the potential long-term ocular or systemic side-effects of atropine usage. Several anticholinergic drugs and other medications with anticholinergic properties have been associated with central side-effects adversely affecting cognitive function.^91^ Although this is particularly true for elderly people with high anticholinergic load, prolonged exposure to even mild anticholinergics during midlife can put one at increased risks in old age.^92^ Given that some clinicians in East Asia advocate continuous treatment of myopic children with low-dose atropine till late adolescent period, after which myopia generally stabilizes, the long-term central effects of extended exposure to atropine, also an anticholinergic, in early life should be investigated thoroughly. Taken together, there is a need to study the mechanisms of action of atropine in preventing myopia development to develop targeted therapies with enhanced efficacy and minimal short-term/long-term adverse effects. There is also a necessity to identify new druggable targets and develop alternative therapeutic strategies for myopia control in the subset of patients who fail to respond to atropine treatment.
